# Oil-based and oil-free formulations for enhancing cannabidiol bioavailability

**DOI:** 10.1186/s42238-025-00371-y

**Published:** 2025-12-02

**Authors:** Petr Jelínek, Anežka Klouček, Ashley Hannah George, Hynek Housar, Petr Kozlík, Tomáš Křížek, Pavel Ryšánek, Martin Šíma, Ondřej Slanař, Miroslav Šoóš

**Affiliations:** 1 Department of Chemical Engineering, Faculty of Chemical Engineering, University of Chemistry and Technology, Prague, Czech Republic; 2 Institute of Pharmacology, First Faculty of Medicine, Charles University, General University Hospital in Prague, Prague, Czech Republic; 3 Department of Analytical Chemistry, Faculty of Science, Charles University, Prague, Czech Republic

**Keywords:** Cannabidiol, Emulsion, Nanoparticles, *In vivo* study, Bioavailability

## Abstract

**Background:**

Cannabidiol (CBD) exhibits therapeutic potential due to its analgesic, anxiolytic, anti-inflammatory, and anticonvulsant effects. However, its oral bioavailability is limited by poor water solubility and extensive first-pass metabolism. Formulation strategies such as oil-based emulsions and oil-free particles may overcome these limitations by enhancing solubilization and promoting lymphatic absorption. This study aimed to evaluate the effects of oil droplet and particle size, and surfactant concentration on CBD bioavailability.

**Methods:**

CBD emulsions were produced using membrane emulsification, high-pressure homogenization, while particles were produced via solvent emulsification–evaporation method. Physicochemical properties were assessed using microscopy and light-scattering techniques. In a randomized, cross-over study, male Wistar rats (*n* = 75) received single oral doses of ten test formulations, while a CBD solution in sunflower oil served as the reference. Serum concentrations were determined using validated UHPLC–MS/MS. Pharmacokinetic parameters (AUC_last_, C_max_, T_max_) were estimated by non-compartmental analysis and statistically compared using ANOVA.

**Results:**

All tested formulations enhanced CBD absorption relative to the reference, CBD in sunflower oil. Among emulsions, droplet size significantly influenced bioavailability: the 16 μm formulation yielded the highest exposure, with AUC_last_ and C_max_ values reaching 291% and 455% of the reference, respectively. Both sunflower and sesame oil emulsions enhanced bioavailability against the oil solution, though sunflower oil showed a slight advantage. Oil-free nanoparticles and microparticles also improved absorption due to their amorphous character, with size exerting minimal effect. Higher concentrations of Tween 20 accelerated absorption but reduced overall exposure, while an excess of lecithin decreased bioavailability.

**Conclusions:**

CBD bioavailability can be substantially enhanced by formulation design. Medium-sized emulsions (≈ 16 μm) provided the most pronounced improvement, while oil-free particles offered additional but less size-dependent benefits. Excessive surfactant (Tween 20) or lecithin content negatively impacted systemic exposure, underscoring the need for balanced formulation strategies. These findings contribute to the understanding of oral delivery of lipophilic compounds and support the rational development of optimized CBD formulations for therapeutic applications.

**Supplementary Information:**

The online version contains supplementary material available at 10.1186/s42238-025-00371-y.

## Introduction

Cannabidiol (CBD) is a chemical compound found in the cannabis plant. It is one of over 100 phytocannabinoids that have been identified in *Cannabis sativa* (Kinghorn et al. 2017), and it has gained significant attention in recent years for its potential therapeutic benefits and its non-psychoactive nature. CBD affects the endocannabinoid system and modulates the activity of many other cellular receptors, including serotonin receptors (5-HT_1A_) or transient receptor potential vanilloid channels (TRPV) (Britch et al. 2021). Thus, it has been studied for its potential therapeutic properties, which include pain relief, anxiety reduction, anti-inflammatory effects, and anti-seizure properties. Due to its complex mechanism of action, CBD and other cannabinoids have been studied for their pharmacological potential in metabolic (Wicinski et al. 2023), cardiovascular (Kicman and Toczek 2020), and oncology diseases (Tomko et al. 2020).

CBD has become an attractive option for people seeking the potential benefits of cannabis without altering their mental state, resulting in a continuously growing market. As a result, CBD is currently available in various forms, including oils, tinctures, capsules, edibles, topicals (creams and balms), and even inhaled products like vaporizers. However, the Food and Drug Administration (FDA) and European Medicines Agency (EMA) have approved only one CBD-based product. Epidiolex (oral solution) is the first approved drug for the treatment of seizures in rare forms of epilepsy, while Sativex (oromucosal spray), the other approved cannabis-based treatment for spastic symptoms, contains both CBD and tetrahydrocannabinol (THC). CBD research is ongoing, and numerous studies have been conducted to investigate potential benefits and risks.

Oral administration is the most preferred route for drug delivery. Nevertheless, the high lipophilicity of CBD is a significant issue for practical applications; CBD is classified as Class II according to the Biopharmaceutics Classification System (BCS) (Stella et al. 2021). Low water solubility leads to low bioavailability, resulting in ineffective concentration in serum, especially after oral administration. Furthermore, first-pass hepatic metabolism additionally reduces CBD concentration in plasma (Benet et al. 2011). In contrast with the portal vein-to-liver pathway, the lymphatic system transports substances directly to the systemic circulation. In brief, consumed fats are broken down into fatty acids and monoglycerides. These are absorbed into enterocytes, where fats are reformed. The newly formed triglycerides are mixed with cholesterol and other components creating lipid particles called chylomicrons. The chylomicrons are then released into the lymphatic drainage system and further into the bloodstream (Zhang et al. 2021).

Thus, a potential method to increase CBD oral bioavailability is co-administration with a high-fat meal, as Zgair et al. (2017) reported. They compared CBD and THC absorption after oral administration, concluding that the concentration in plasma for the fat-containing formulation was three times higher than the fat-free formulation in rats. The concentration in plasma and lymph was investigated after oral administration of fat-containing formulation. CBD concentration in lymph fluid was 250-fold higher than in plasma. Moreover, they showed cannabinoids to have an immunomodulatory effect, affecting lymphocytes in the lymphatic system. Thus, fat-assisted administration showed the potential to improve the treatment of multiple sclerosis and other autoimmune disorders.

To further increase bioavailability, lecithin—a naturally occurring phospholipid and commonly used emulsifier—plays a crucial role in enhancing lipid digestion and drug absorption. Robert et al. (2020) summarized current knowledge of how vegetable lecithins affect lipid digestion and metabolism. Next, Couedelo et al. (2015) tested the effect of common industrial food emulsifiers on lipolysis in vitro and lymphatic absorption in rats. Lipolysis and lymphatic absorption were increased with lecithin-stabilized emulsion compared to plain flaxseed oil and emulsions stabilized with Tween 80 and sodium caseinate. Further support for this observation comes from a meta-analysis of various highly hydrophobic drugs, which found an 18.5% enhancement in the overall bioavailability for peroral phospholipid-based solid formulations (Fong et al. 2015). Given its dual role as a surfactant and bioavailability enhancer, lecithin was selected as a key excipient in our formulation strategy.

In accordance with the literature, the bioavailability enhancement was observed in our recent study, where we showed a positive effect of orally administered CBD in the lecithin-stabilized oil-in-water (O/W) emulsions on symptoms of rheumatoid arthritis induced in rats (Jelínek et al. 2024). However, the formulation was evaluated as a whole, and the specific contribution of individual formulation parameters to bioavailability was not investigated in detail. Moreover, to the best of our knowledge, a comprehensive comparative study of various formulations and parameters is lacking in the literature.

Building on previous findings that oil-based formulations and lecithin-stabilized emulsions enhance the bioavailability of lipophilic drugs, we designed a comparative study focused on the effect of individual parameters to address this gap. The parameters selected—formulation composition, particle/droplet size, surfactant concentration, and oil type—were chosen based on their mechanistic relevance to lipid digestion, emulsification efficiency, and lymphatic transport, which are critical for improving the absorption of lipophilic compounds such as CBD. Particle size influences dissolution rate and gastrointestinal absorption, especially for poorly water-soluble drugs (Macedo et al. 2024). Droplet size in emulsions affects interfacial area and digestion kinetics, with smaller droplets promoting faster lipid digestion and more efficient uptake (Salvia-Trujillo et al. 2013). Surfactant concentration modulates micelle formation and solubilization capacity, which are essential for maintaining CBD in a bioavailable form during digestion (Holzem et al. 2024). Oil type determines the fatty acid profile and influences chylomicron formation, which facilitates lymphatic transport and systemic absorption of lipophilic drugs (Feng et al. 2022). By systematically varying these parameters while maintaining consistent experimental conditions, we aimed to identify their individual and combined effects on oral CBD bioavailability in vivo.

## Materials and methods

### Materials

CBD (98.9 wt%) was purchased from PharmaHemp (Slovenia). Sunflower oil, a standardized pharmaceutical quality product, was purchased from Fagron (Czech Republic). Soybean lecithin (≥ 97%) was purchased from Carl Roth (Germany), Tween 20 (TW20) and sesame oil from Sigma-Aldrich (Germany), and diethyl ether (DEE) from Penta (Czech Republic). Syringe filters 1 μm (glass fiber) and 450 μm (nylon) were purchased from VWR International (USA), Sefar NITEX mesh (50 μm, 100 μm) from Sefar (Switzerland), and Shirasu Porous Glass (SPG) membrane (CP-20 K-50 N) from SPG Technology (Japan). For an in vivo study, isoflurane (IsoFlo, 250 mL; Zoetis/Pfizer, Czech Republic), ketamine (Narkamon, 100 mg/mL, inj. Sol.; Bioveta, Ivanovice na Hané, Czech Republic) and xylazine (Rometar, 20 mg/mL, inj. Sol.; Bioveta) were used for anesthetization. Heparin (Heparin Léčiva, inj. Sol., 1 × 10 mL/50 KU; Zentiva, Czech Republic), Carbethopendecinium bromide (Ophthalmo-Septonex, eye ointment, 1 × 5 g; Zentiva) and ketoprofen (Ketodolor, inj. Sol.; 100 mL; LeVet Pharma b.v., Netherlands) were used as a perioperative anticoagulant, antiseptic and analgesic treatment, respectively. The combination of Mebezonium iodide, Embutramide, and Tetracaine hydrochloride (T 61, inj. Sol.; Intervet International, B.V., Netherlands) was used for euthanasia.

### Emulsions

The emulsion was prepared in two steps. At first, the coarse emulsion was prepared using the oil (3 g of sunflower or sesame oil), which was added to the lecithin (1.5 g) and CBD (112.5 mg). The mixture was stirred with a magnetic stirrer for an hour. Then, deionized (DI) water (12 mL) was added and gently mixed. Second, the coarse emulsion was further processed by membrane homogenization or high-pressure homogenization (HPH) to obtain droplet sizes suitable for this study. In brief, coarse emulsion was pushed through the membrane (nylon mesh, syringe filter, or SPG membrane) using a syringe pump (PHD 4400; Harvard Apparatus, USA). In the case of HPH, the coarse emulsion was pre-homogenized using high-shear homogenization at 20,000 RPM for 10 min using UltraTurrax T25 (IKA, Germany). Then, HPH (Lab Homogenizer PandaPLUS 2000; GEA, Germany) was performed under 1,700 bar. The emulsion was cooled in a water bath before each cycle.

Prepared emulsions were stored in the fridge before further use. The stability of the samples was confirmed three days post-preparation; however, samples intended for animal experiments were prepared one day prior to administration in order to minimize alterations in size distribution.

The size distribution of formed emulsions was determined via image analysis of pictures collected with an optical microscope (customized system - tube lenses (Resolv4K; Navitar, USA) equipped with objective lenses (Olympus, USA), combined with the high-speed camera (PLD725MU-T; PixeLINK, USA)). At least 500 droplets were measured for volumetric distribution analysis. In the case of micron- and nanosized emulsions, static light scattering (SLS; Mastersizer 3000, Malvern Panalytical, UK) was used to characterize their size.

### Particles

CBD nano- and microparticles were prepared using a solvent emulsification evaporation technique. In general, lecithin and CBD (7.5 mg/g in the final volume) were dissolved in DEE (8 ml) to form the organic phase, which was then directly transferred into a beaker containing demineralized water (20 ml). The Ultra-Turrax was used to homogenize the two immiscible phases. During the emulsification process, samples were kept on ice, and the top of the vessel was sealed to prevent the evaporation of the highly volatile solvent. The final step involved transferring the emulsion to a round-bottom flask to remove DEE by rotary evaporation (45 °C water bath and 700 mbar) until the organic solvent was no longer noticeable in the sample. To investigate the impact of non-ionic surfactant (TW20) on bioavailability, a specific amount of TW20 was added to the nanoparticle suspension to adjust the surfactant concentration to the final values while stirring gently for 15 min. The amount was based on the critical micellar concentration (CMC) value (Mahmood and Al-Koofee 2013). Micron-sized samples were analyzed by SLS, while samples containing sub-micron particles were analyzed using dynamic light scattering (Zetasizer NanoSZ; Malvern Panalytical, UK).

### In vivo animal tests

Male Wistar rats were purchased from Velaz (Prague, Czech Republic). The rats were housed under controlled conditions with temperature (22 ± 2 °C) and humidity (50 ± 10%) with a 12-h light-dark cycle. All animals were provided free water access and a standard granulated diet. The animal study was approved by the Ministry of Education, Youth, and Sports, Czech Republic under No MSMT-26,838/2021-4. Animals were maintained following the Guiding Principles for the Use of Animals at Charles University, First Faculty of Medicine.

The relative oral bioavailability of different CBD test formulations (T1-T10) compared with CBD in sunflower oil solution as a reference (R1-R10) was tested in a randomized, single-dose, two-sequence, two-period, open-label, cross-over comparative bioavailability study.

All animals underwent surgery two days before the experiment. During the surgery, a polyurethane catheter (3Fr; Intech Laboratories, Plymouth Meeting, the United States) was inserted into the external jugular vein. The catheter allowed repeated blood sampling. The surgery was performed under general anesthesia using isoflurane (2.5–5.5%), followed by intramuscular administration of ketamine (100 mg/kg) and xylazine (5 mg/kg). Heparin was used as a perioperative anticoagulant, and Carbethopendecinium bromide as an antiseptic treatment. Ketoprofen (5 mg/kg) was administered subcutaneously immediately after surgery. The rats were euthanized (T61^®^) at the end of the experiment.

On the third day after the cannulation, rats were divided into groups. Each group received one of the tested formulations and the reference. Animals were fasted for 4 h before and after dosing with free access to water. The administration of 1 mL of reference or test formulations (all containing 7.5 mg of CBD) was performed via oral gavage. Blood samples (120 µL) were collected from each rat pre-dose (second period) and at 1, 2, 3, 4, 5, 6, 8, 10, and 24 h after the dosing (both periods). The volume of blood sampled was replaced by an equal volume of saline and 50 µL of heparinized saline flush (1000 IU/mL). Blood samples were centrifuged at 2000 g, 4 °C, for 10 min. The separated serum was stored at −80 °C until further analysis. The washout period of 72 h was applied between the first and the second dosing.

### Analytical methods

CBD concentration in serum was determined by sensitive UHPLC-MS/MS (ultra-high performance liquid chromatography-tandem mass spectrometry) with an isotopically labeled internal standard, following our previously described protocol (Jelínek et al. 2024). The method was validated according to the requirements of the European Medicines Agency (EMA) Guideline on bioanalytical method validation in terms of linearity, lower limit of quantification (LLOQ), upper limit of quantification (ULOQ), accuracy, precision, selectivity, recovery, carry-over effect, matrix effects, robustness, dilution integrity, and stability of quality control (QC) samples and clinical patient samples, and has been demonstrated to be suitable for its intended purpose (European Medicines and Agency 2022). The UHPLC-MS/MS method with one-step sample preparation provided high sensitivity and high sample throughput (Jelínek et al. 2024).

### Data analysis and statistics

Actual sampling times were used for all pharmacokinetic calculations, while scheduled sampling times were used only for plotting pharmacokinetic profiles. CBD serum concentrations were dose-normalized to 25 mg/kg before calculations. Pharmacokinetic analysis was performed in a non-compartmental model using Phoenix WinNonlin^®^ (Certara, Princeton, the United States). The area under the curve from time 0 to the last sampling time (AUC_last_) was calculated using the linear-trapezoidal method. The maximum serum concentration (C_max_) and the corresponding time (T_max_) were directly observed. The natural logarithmic transformation of C_max_ and AUC_last_ was used for all statistical inference. C_max_ and AUC_last_ are expressed as geometric means ± 90% confidence interval (CI), while T_max_ is presented as a median ± interquartile range (IQR). Mean pharmacokinetic profiles ± standard deviation (SD) were used for graph plotting. To report bioavailability of prepared CBD formulation with respect to sunflower oil solution reference, mean values of AUC_last_, C_max_ and T_max_ were reported as the ratio of our test formulations and reference (T/R).

AUC_last_ and C_max_ values were compared using a standard ANOVA model in accordance with EMA guidelines (European Medicines Agency 2010) using a 90% confidence interval for the ratio of geometric least squares means. The fixed factors included in this model were the effects of subject, treatment, period, and sequence. A comparison of median T_max_ value and mean concentration in each sampling time was performed using the Wilcoxon matched-pairs signed rank test and paired t-test, respectively, within GraphPad Prism version 9.1.0. (GraphPad Software, San Diego, CA, USA). Statistical significance was considered at *P* ≤ 0.05.

## Results and discussion

### Lecithin-stabilized emulsions

Since the impact of droplet size on the bioavailability of CBD has not been systematically investigated, five different-sized emulsions were prepared for testing in the pharmacokinetic study. Unfortunately, the previously used high-shear method (Jelínek et al. 2024) was unsuitable for preparing narrow droplet size distributions. Hence, membrane emulsification and high-pressure homogenization were employed. Furthermore, optimization of process parameters was necessary to develop a procedure suitable for the preparation of emulsions containing CBD for rat administration (see Table 1).

**Fig. 1:**
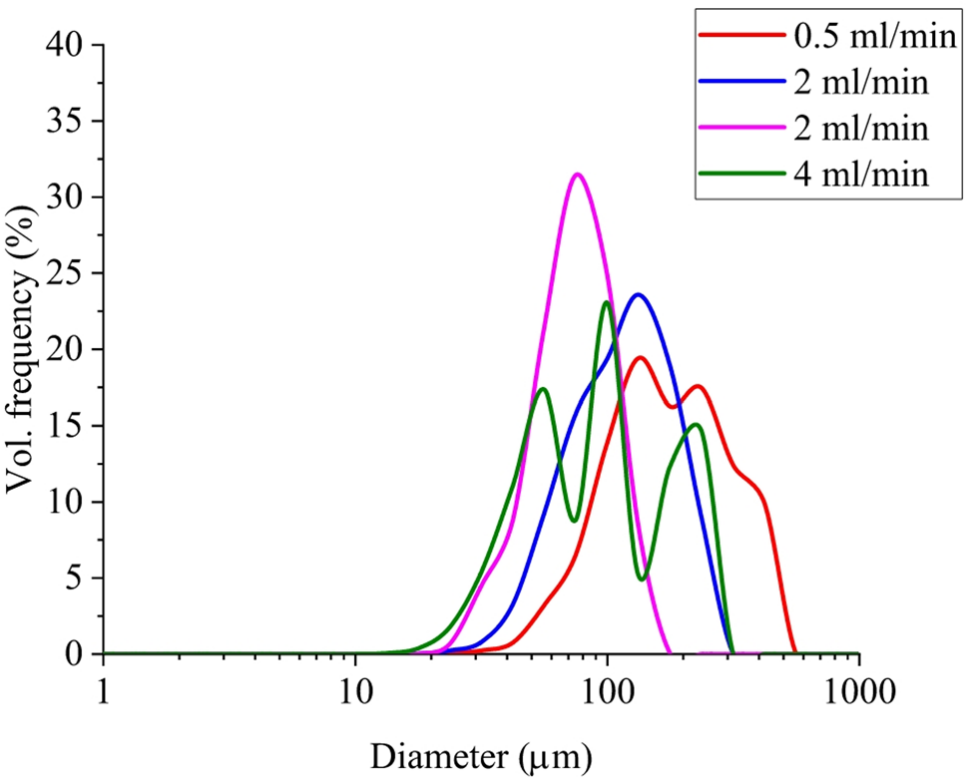
Size distribution of emulsions prepared via membrane emulsification using a 100 μm mesh at different flow rates. The blue line represents the distribution of droplets after one pass, while the magenta line shows the distribution of droplets after two passes through the apparatus, in both cases flow rate of 2 mL/min was used

An example of prepared droplet populations prepared by membrane emulsification using a mesh with a size of 100 μm coupled with various flow rates is shown in Fig. 1. As can be seen, the flow rate had a substantial impact on the final droplet size distribution with a mean diameter in the range from 70 to 200 μm. In particular, an increase of the flow rate from 0.5 mL/min to 2 mL/min reduced the droplet diameter. However, a further increase to 4 mL/min caused the formation of a multimodal distribution. In contrast, when using an intermediate flow rate (i.e., 2 mL/min) combined with an increased number of passes, the size was reduced while keeping the low polydispersity of the sample. Therefore, the flow rate of 2 mL/min, coupled with two membrane passes, was used to produce a relatively narrow distribution within the targeted range. Such conditions were used to prepare the corresponding emulsion in the pharmacokinetic study (see T4 in Table 1).

**Fig. 2:**
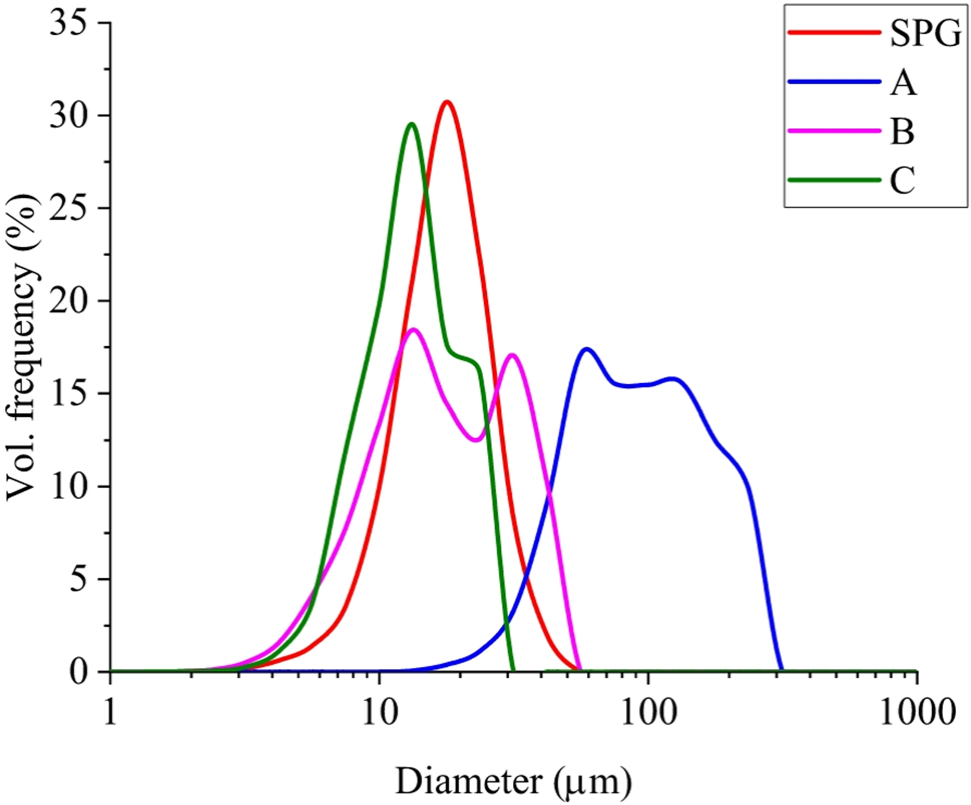
Size distribution of emulsions prepared via membrane emulsification. (red line) droplet distribution prepared by SPG glass membrane (T3). **A**- one pass through one layer of mesh with 5 μm openings at 2mL/min; **B**- emulsion A pushed through two layers of mesh with 5 μm openings at 2mL/min; C- emulsion B pushed through two layers of mesh with 5 μm openings at 2mL/min

To produce an emulsion with a mean size of around 10 μm, we tested a procedure using a 5 μm nylon mesh. As shown in Fig. 2, by combining multiple layers of nylon mesh with multiple passes, we were able to prepare an emulsion in the range, however, showing multiple populations.

Further increase in the number of membrane layers and passes did not lead to an additional decrease in size and improvement of polydispersity. Hence, the SPG membrane module was employed. The SPG membrane system (50 μm, hydrophilic), operated at a flow rate of 1 mL/min, yielded a narrow size distribution within the targeted range. This procedure was used for the preparation of the emulsion administered in rats (T3 in Table 1).

**Fig. 3:**
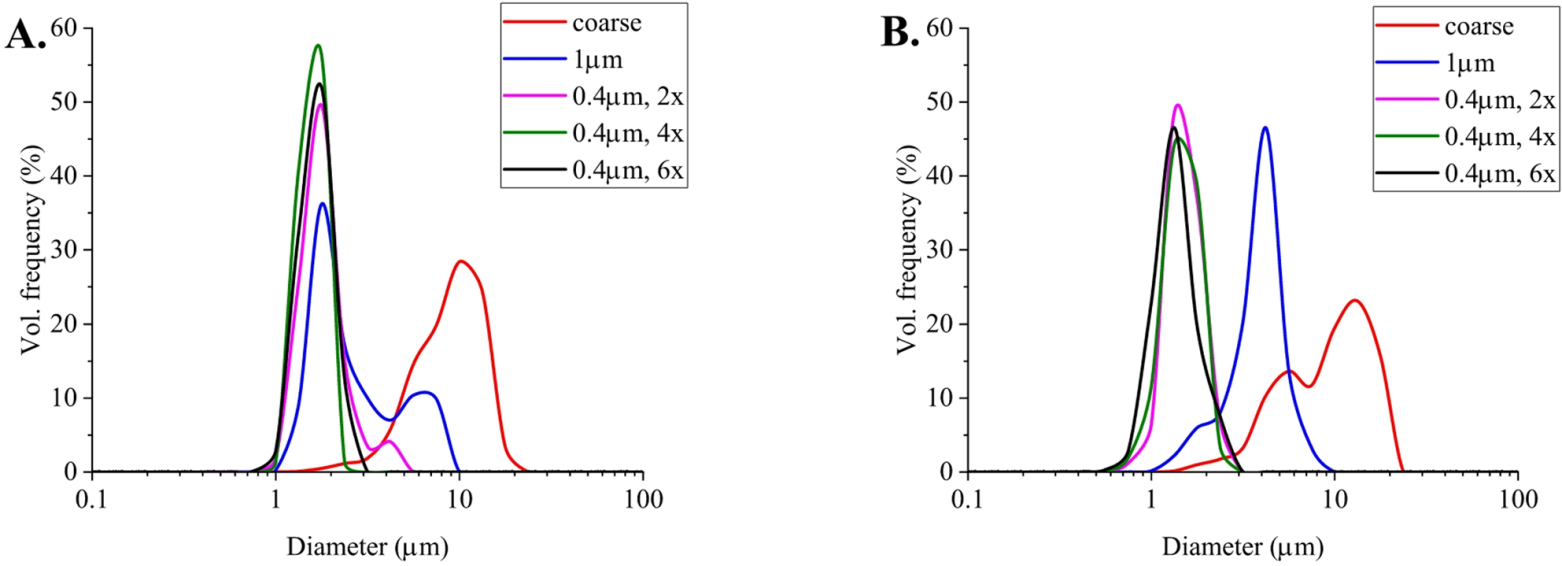
Size distribution of emulsions prepared via membrane emulsification with sunflower oil (**A**.) and sesame oil (**B**.). Legend indicates the used membrane porosity and number of passes

To prepare an emulsion with an average diameter of 1 μm, we used syringe filters with pore sizes of 1 and 0.45 μm. As shown in Fig. 3A, a 1 μm syringe filter resulted in a rather broad droplet size distribution with minimum droplet sizes near 1 μm. Unfortunately, we were unsuccessful in reducing droplet size polydispersity. Therefore, a 0.45 μm syringe filter was used to further reduce the size of large droplets. Multiple passes were tested to observe the effect on size distribution. As seen in Fig. 3A, this approach allowed us to achieve a rather narrow droplet size distribution with the desired average diameter and low polydispersity. Three passes were found to be a compromise between the width of the produced droplet size distribution and sample loss during the process. Furthermore, the same procedure was applied to sesame oil with comparable results (see Fig. 3B). Such conditions were used for the preparation of the emulsions tested in rats (T1, T2 in Table 1).

**Fig. 4:**
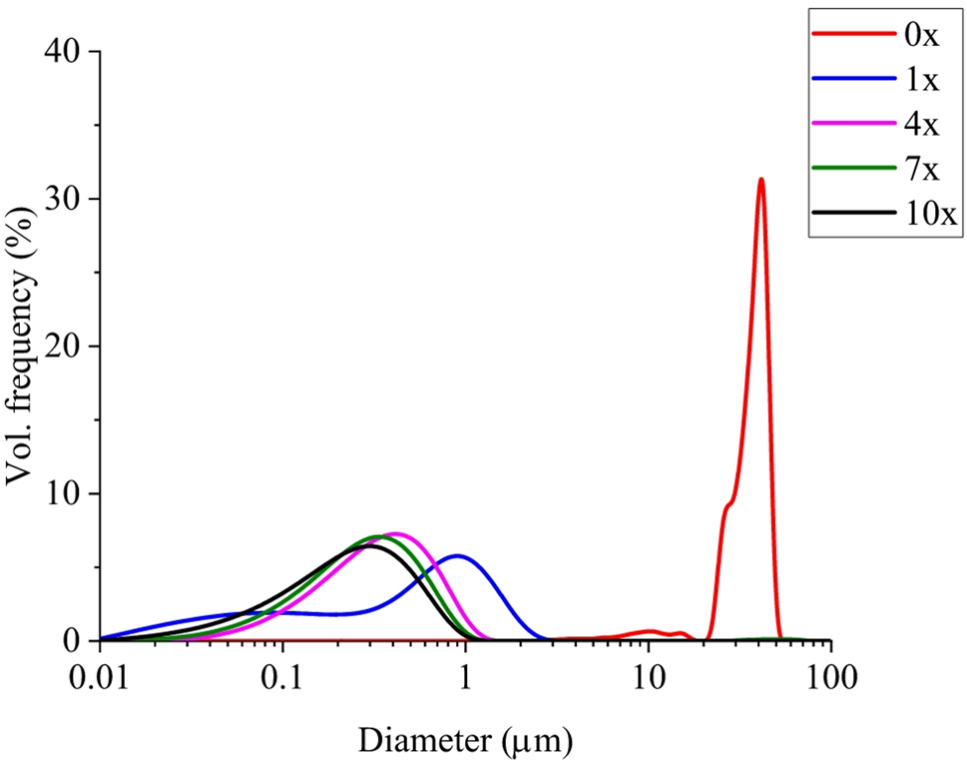
The effect of a number of passes through HPH on droplet size of emulsion. Legend indicates the number of passes

HPH was employed to obtain a submicron emulsion, as variations in membrane emulsification parameters did not result in droplets of submicron range. An example of a prepared nanoemulsion using a various number of passes through HPH is presented in Fig. 4. As can be seen, droplet size was significantly reduced with a large fraction of the submicron particles just after a single pass through HPH. After four passes, the droplet distribution becomes monomodal with an average diameter of about 400 nm. Further increase in the number of passes through the HPH instrument did not result in a significant reduction in droplet size. Moreover, the heat generated during homogenization may adversely affect the stability of CBD, potentially leading to its decomposition and destabilization of the emulsion system, despite the sample being cooled in an ice bath prior to each cycle. Thus, seven passes through HPH at 1,700 bar were used to prepare the sample used in the pharmacokinetic study (T5 in Table 1).

The resulting mean droplet size and polydispersity index of emulsion samples administered to rats are shown in Table 1. Please note, that these samples were prepared according to the optimized procedure described above and only with the necessary amount required for rat administration. As illustrated, all samples prepared by membrane emulsification exhibited narrow droplet distribution with mean sizes of 1, 16, and 75 μm, all with relative spans below 0.9. In contrast, the HPH method produced a significantly broader distribution with a mean size of 250 nm and a relative span of 1.9.

**Table 1:** Preparation parameters, size, and relative span of CBD emulsion samples used in bioavailability study

Sample	Oil	Oil (wt%)	Lecithin (wt%)	Emulsification method	Mean diameter (µm)	Relative span
T1	Sunflower	18	9	Membrane	1.1	0.83
T2	Sesame	18	9	Membrane	1.1	0.84
T3	Sunflower	18	9	Membrane	16.0	0.83
T4	Sunflower	18	9	Membrane	75.0	0.89
T5	Sunflower	18	9	HPH	0.25	1.93

### Lecithin-stabilized particles

As an alternative to oil-based emulsions, the solvent emulsification evaporation method was utilized to develop stable CBD nano- and micron-sized particle suspensions. The optimal concentration of lecithin was found to be 1.25 wt%, as lower concentrations resulted in the instability of the suspension, specifically the formation of large aggregates that settled at the bottom of the beaker. Therefore, all formulations except T10 adhered to the optimal lecithin concentration, so the particle size was influenced only by the homogenization parameters – RPM and time. Developing CBD nanoparticles of approximately 200 nm in diameter with a narrow size distribution required homogenization at 17,000 RPM for 40 min. Micron-size CBD formulations (T7, T8, and T9) were produced by homogenization at 12,800 RPM for 25 min, showing monomodal size distributions and high reproducibility, even with the addition of a co-surfactant (T8 and T9). The addition of TW20 to the already prepared microparticles did not affect their mean diameter, although there was a slight decrease in the zeta potential. Formulation T10 contained particles with a mean particle size of about 3 μm and acceptable polydispersity. Introducing a large amount of lecithin required an increasing the organic phase volume to dissolve all components. Despite our efforts, we were unable to prepare stable particles with a mean diameter of above 10 μm. Lower RPM and varying volumes of added organic solvent resulted in unstable suspensions that were not characterized. Detailed information about composition, process parameters, resulting mean diameter, distribution span, and zeta potential is summarized in Table 2. As can be seen, an increase of the particle diameter is connected with an increase of the polydispersity, reaching a value comparable to the HPH method. In all cases of nanoparticles, the measured zeta potential is comparable, suggesting good stability of nanosuspensions.

**Table 2:** Preparation parameters, final size, and zeta potential of CBD nanoparticle samples used in bioavailability study

Sample	Lecithin concentration (wt%)	Relative concentration to CMC of TW20	DEE volume^a^ (mL)	RPM (×1000)	Time^b^ (min)	Mean diameter (µm)	Relative span	Zeta potential (mV) ± SD
T6	1.25	-	8	17	40	0.223	1.11	−56.4 ± 1.5
T7	1.25	-	8	12.8	25	1.05	1.71	−64.6 ± 0.3
T8	1.25	10	8	12.8	25	1.15	1.80	−62.8 ± 1.3
T9	1.25	100	8	12.8	25	1.17	1.86	−52.8 ± 0.2
T10	10	-	16	20	20	3.31	2.56	−64.4 ± 1.5

^a^final formulation volume: 20 ml, ^b^time of homogenization

### Pharmacokinetic study

Seventy-five rats (350–500 g) completed the cross-over study. They were divided into eleven groups (*n* = 6–7). The cross-over study design reduces the number of animals needed for the study and ensures high-quality data within the study groups (Královicová et al. 2022). Plain CBD solution in sunflower oil was selected as the reference for all study groups. Bioavailability results are presented as a bar chart, depicting the ratio of geometric mean values of C_max_ and AUC_last_ relative to the reference formulation (T/R). Error bars indicate the ± 90% CI. The complete summary of pharmacokinetic parameters (AUC_last_, C_max_, T_max_) is reported in the Supplementary material (Table-SI 1).

#### Oil type

One of this study’s aims was to test the effect of carrier oil on bioavailability. Various oils and triglycerides were tested in the literature. For example, Feng et al. (2021b) compared complex sesame oil with separated triglycerides. Pre-digested and purified lipids did not reach the pharmacokinetic parameters of natural sesame oil. Moreover, sesame oil, a main component of Epidiolex, was selected as a potential candidate to enhance bioavailability in the form of an emulsion in this study. In the following work, Feng et al. (2022) tested the effect of carrier oil on CBD bioavailability. They observed a slight difference in CBD serum concentration between various vegetable oils when administered orally. Nevertheless, neither mentioned publications observed a significant difference in the plasma CBD concentration between the oils.

**Fig. 5:**
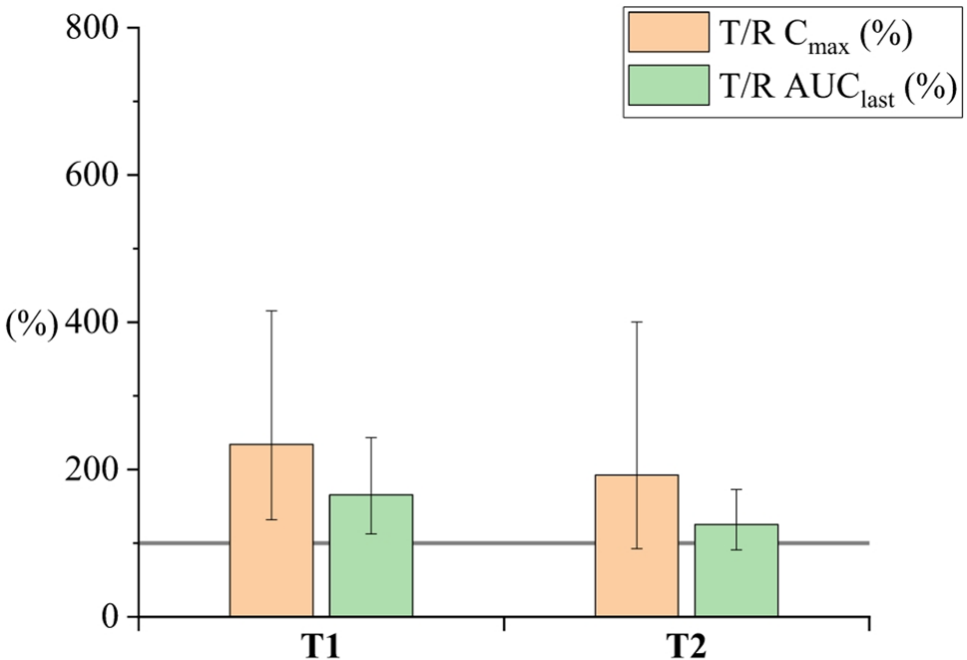
Relative C_max_ and AUC_last_ for lecithin emulsions, comparison of different oils: T1- sunflower oil-based emulsion, T2- sesame oil-based emulsion. The gray line represents 100%, indicating the oral bioavailability of CBD in the reference sunflower oil solution. C_max_, AUC_last_ reference ratios are given as geometric means (± 90% CI). Values are shown in the supplementary material

Therefore, in the present work, sunflower oil-based (T1) and sesame oil-based (T2) emulsions were prepared via membrane emulsification. Both samples had the same size (about 1 μm) and the same composition (apart from the oil type). The measured pharmacokinetic parameters of the sunflower emulsion (T1) were almost doubled compared to the reference sunflower solution. The sesame-oil-based emulsion (T2) has slightly lower bioavailability than the same-sized sunflower-oil-based emulsion (T1), even though the difference between both emulsions did not reach statistical significance (Fig. 5). For further experiments, sunflower oil was chosen.

#### Droplet size

In our recent study, we demonstrated increased CBD bioavailability for an oil-based formulation (Jelínek et al. 2024); however, the impact of droplet size on bioavailability was not investigated in detail. To address this, we prepared a series of emulsion samples with varying average sizes but with identical composition, allowing us to isolate the effect of size. This approach enabled us to evaluate the relationship between emulsion droplet size and CBD bioavailability, and to determine whether differences exist between oil-based formulations (emulsions) and oil-free formulations (nano- and microparticles).

**Fig. 6:**
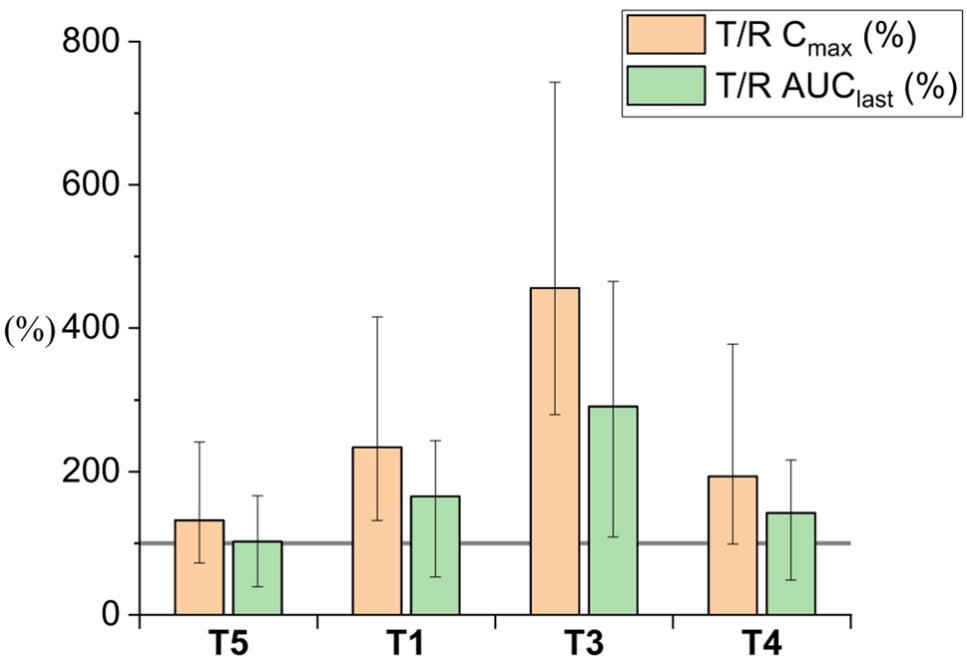
Relative C_max_ and AUC_last_ for lecithin emulsions with different sizes. Samples are sorted according to droplet size increasing from the left. The gray line represents 100%, indicating the oral bioavailability of CBD in the reference sunflower oil solution. C_max_, AUC_last_ reference ratios are given as geometric means (± 90% CI). Values are shown in the supplementary material

As can be seen in Fig. 6, neither C_max_ nor AUC_last_ dropped below the reference values for all measured emulsions. Furthermore, we observed the highest bioavailability (291.1%) for the emulsion with 16 μm-sized droplets (T3). The C_max_ and the AUC_last_ were increased 4.7-fold and 2.8-fold, respectively, compared to the reference (sunflower oil solution). These values are higher compared to those measured in our previous work (Jelínek et al. 2024) considering emulsion distribution was very polydisperse covering range from 10 to 100 μm, where the AUC and C_max_ were 3811 ng/mL.h and 860 ng/mL, respectively, compared to those obtained in this work with AUC 4357 ng/mL.h and C_max_ 888 ng/mL for the T3 formulation with narrow distribution. We are aware of the limitations for direct comparison of these formulations, wherein, in a crossover study, each subject acts as its own control. However, since both studies were performed in the same institution, we do not expect any variations in environmental conditions (e.g., temperature, humidity) or procedural details (e.g., handling, sampling, and analytical technique). Therefore, we conclude that a simple comparison of pharmacokinetic parameter values is possible.

Furthermore, when comparing bioavailability measured for the emulsion T4 with the largest droplet sizes, our results align with the literature data. Feng et al. (2021a) mixed sesame oil with medium-chained triglycerides (MCT) and various surfactants to increase the bioavailability of CBD in rats. The results of in vitro lipolysis showed increased CBD amount in the micellar phase compared to sesame oil, while the in vivo test showed no significant difference in the bioavailability of CBD, suggesting a negligible effect of MCT and surfactants when administered as a bulk phase.

On the other hand, low bioavailability for submicron formulation (T5) at the level of 102.6% compared to the reference solution is surprising. Zheng et al. (2020) measured a doubled AUC of carnosic acid (C_max_ was comparable) for lecithin-based nanoemulsion compared to suspension, indicating that the combination of the nanosized oil-based formulation and the lecithin presence could be beneficial for CBD bioavailability enhancement. The difference between the mentioned paper and our nanoemulsion can be related to formulation composition, especially the amount of lecithin (only 3 wt% in the case of Zheng et al.) and the type of oil. Further differences in bioavailability can be related to much lower partition coefficients of carnosic acid compared to CBD.

#### Particle size

**Fig. 7:**
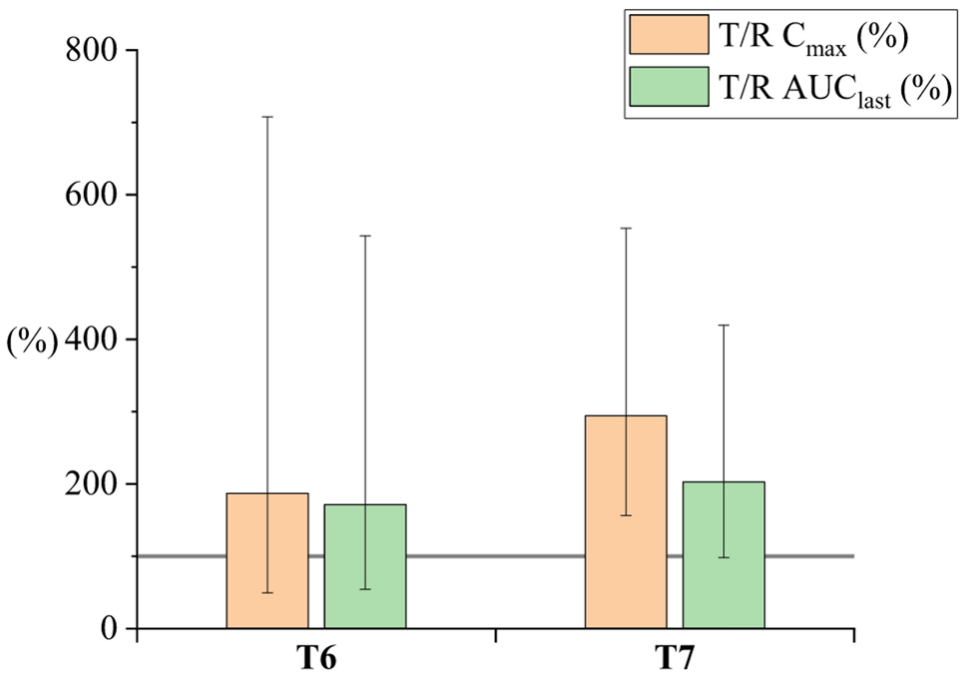
Relative C_max_ and AUC_last_ for CBD particles with different sizes (T6-nanoparticles, T7-microparticles). The gray line represents 100%, indicating the oral bioavailability of CBD in the reference sunflower oil solution. C_max_, AUC_last_ reference ratios are given as geometric means (± 90% CI). Values are shown in the supplementary material

The presence of oil impacts the uptake of poorly soluble drugs when administered orally, as shown by Zgair et al. (2016). Therefore, oil-free formulations were prepared (Table 2) and tested. Since the size of particles impacts the bioavailability, samples (T6, T7) with different sizes were prepared by optimizing preparation parameters according to Table 2. As shown in Fig. 7, both samples exhibited a trend toward higher absorption than the reference, most likely due to the amorphous character of the prepared micro- (T7) and nanoparticles (T6) (see X-ray diffractogram in Figure-SI 1). Furthermore, an amorphous character is also responsible for the insignificant difference between T6 and T7, even though they have different mean diameters, i.e., 220 nm vs. 1 μm. In contrast, when particles are crystalline, particle size would have a substantial impact as demonstrated by Xia et al. (2010), who measured higher bioavailability for smaller particles when comparing various sizes (from 20 μm to 200 nm) of crystalline nitredipine.

#### Surfactant concentration

Regarding the effect of surfactants on in vitro membrane permeation, Amidon et al. (1982) showed that the membrane permeability of progesterone is decreased when a higher amount of surfactant is present in in vitro tests. The effect of reduced permeability was observed for all tested surfactants. Moreover, Volkova et al. (2021) observed increased solubilization of poorly soluble drugs using various excipients. Improved solubilization led to a decrease in enzalutamide and apalutamide permeation. The lowest permeation was shown in samples solubilized by a surfactant. Hence, two sets of particle samples were prepared to verify the observed adverse effect of surfactants in vivo.

**Fig. 8:**
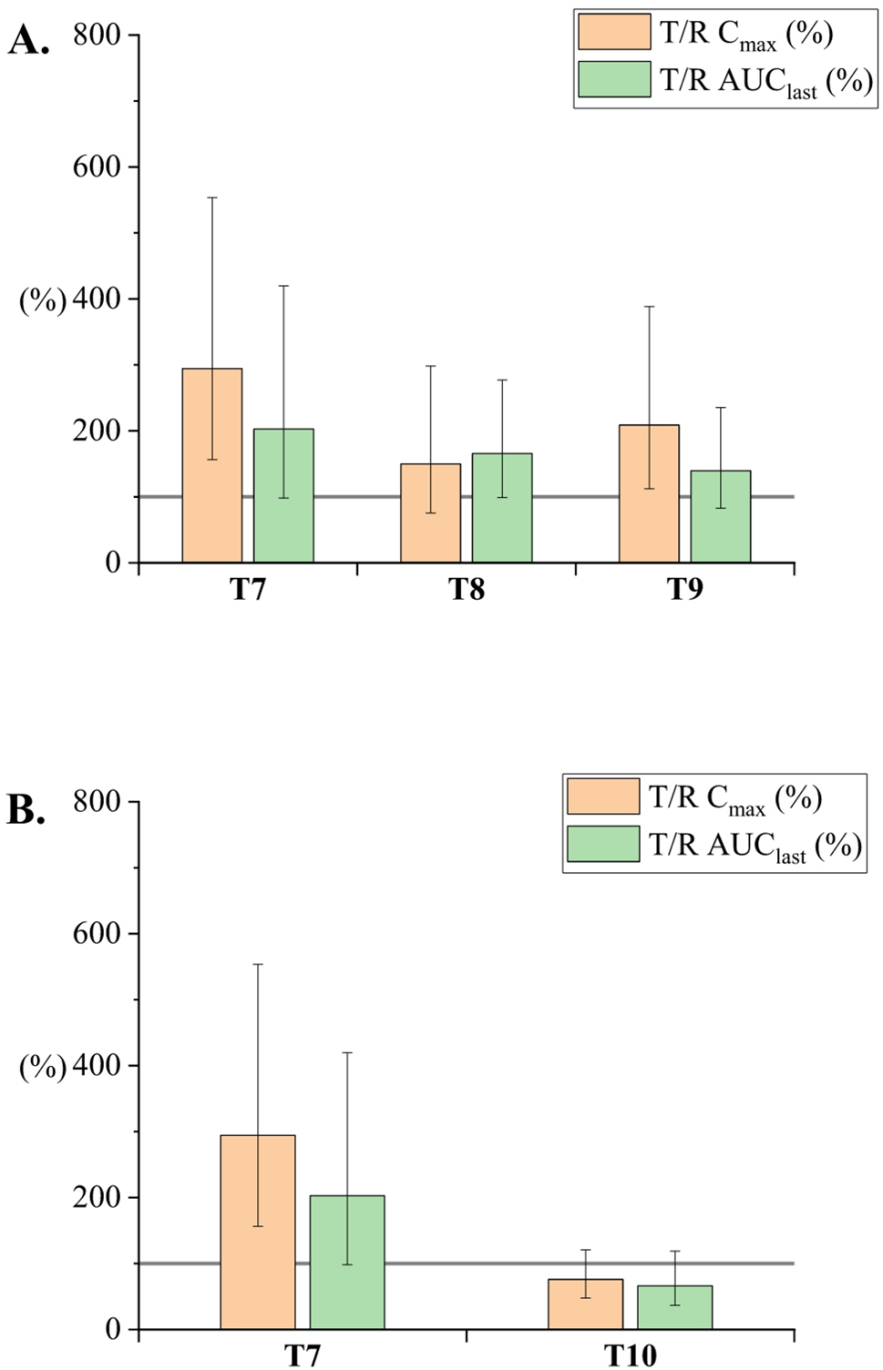
Relative C_max_ and AUC_last_ for CBD nanoparticles. (**A**) increasing concentration of TW20 from T7 to T9, (**B**) 10-fold higher concentration of lecithin in T10. The gray line represents 100%, indicating the oral bioavailability of CBD in the reference sunflower oil solution. C_max_, AUC_last_ reference ratios are given as geometric means (± 90% CI). Values are shown in the supplementary material

In particular, a 10-fold amount of TW20 (T8) and a 100-fold amount of TW20 (T9) relative to TW20 CMC were added to the CBD microparticle suspension according to Table 2. As shown in Fig. 8A, formulations T8 and T9 showed increased bioavailability compared to the reference. Formulation T9 led to a statistically significant C_max_ increase and a significant T_max_ shortening 1.93-fold, indicating a more complete and faster absorption. However, the total bioavailability was only 139.5%, indicating a fast elimination rate. Formulation T7 without the addition of TW20 exhibited a significantly higher C_max_ and a trend toward shortening T_max_ by 1.94-fold, increasing the total exposure to the drug with a bioavailability of 202.8%. The difference in AUC_last_ was negligible when comparing T8 and T9. An even more significant reduction was observed with the nanoparticle formulation T10, which contained a higher concentration of lecithin (see Fig. 8B). In this case, AUC_last_ and C_max_ were lower than formulation T7 and lower than the oil solution reference. This observation is consistent with findings reported by Jacobsen et al. (2021), who showed reduced in vitro permeability and lower bioavailability in rats for phospholipid-stabilized Celecoxib dispersion with increasing stabilizer concentration. In summary, these results demonstrate that the presence of additional surfactant negatively affected bioavailability. This aligns with in vitro measurements published in literature (e.g., Amidon et al., Volkova et al., and Jacobsen et al.).

Furthermore, when comparing formulation T10 to T1 and T2, having comparable size and containing an equivalent amount of lecithin, the presence of oil significantly enhanced bioavailability. Unfortunately, a comparative emulsion with a lower lecithin concentration could not be prepared due to limitations in emulsion system stability. In contrast, nanoparticle formulation T7, despite lacking an oil component, demonstrated bioavailability comparable to emulsion formulations T1 and T2, which had similar particle sizes and contained higher amounts of lecithin. This suggests that the amorphous nature of the nanoparticles and the lower lecithin concentration in T7 may confer an advantage in absorption. Thus, the comparable performance of T7 highlights the potential of oil-free, amorphous formulations with reasonable stabilizer content for effective CBD delivery.

## Conclusion

This study investigated the impact of various carrier oils, size of droplets or particles in the formulation, and the amount and type of surfactants on the bioavailability of CBD in rats. Our findings confirmed that sunflower oil-based emulsions significantly enhanced CBD bioavailability compared to a plain solution of CBD in sunflower oil. Sesame oil-based emulsions also improved bioavailability compared to a solution of CBD in sunflower oil; however, they did not outperform sunflower oil-based emulsions.

The study confirmed that the size of the emulsion droplets plays a significant role in bioavailability, with droplets having a mean diameter of 16 μm showing the highest bioavailability, with 291% and 455% increase in AUC_last_ and C_max_ with respect to the reference oil solution. Oil-free formulations exhibited enhanced absorption, due to their amorphous nature and the presence of lecithin; however, particle size did not significantly impact bioavailability in these formulations.

Formulations with higher amounts of TW20 showed increased bioavailability compared to the reference; however, with faster elimination rates and lower overall bioavailability compared to formulations lacking additional surfactants. These results are in line with the available literature. Furthermore, lecithin-stabilized formulation, particularly solid nanoparticles, emerged as potent bioavailability enhancers for CBD. This work contributes to the understanding of how formulation strategies affect the oral bioavailability of hydrophobic compounds, such as CBD, and can be used for the optimization of cannabinoid formulations in the future.

These findings suggest that oil-based and oil-free formulations can enhance CBD bioavailability, offering valuable insights for developing more effective CBD delivery systems.

## Supplementary Information

Supplementary Material 1

## Data Availability

The data supporting the findings of this study are available from the corresponding author upon reasonable request.
