# Supplementary material for: Oil-based and oil-free formulations for enhancing cannabidiol bioavailability
Source: J Cannabis Res. 2025 Dec 2;8:5. doi: 10.1186/s42238-025-00371-y (PMC12777474; doi:10.1186/s42238-025-00371-y)
Supplement: Supplementary file 1 — Supplementary Material 1 [file 42238_2025_371_MOESM1_ESM.docx]

**Oil-Based and Oil-Free Formulations for Enhancing Cannabidiol Bioavailability**

Petr Jelínek^1,a^, Anežka Klouček^2,a^, Ashley Hannah George^1^, Hynek Housar^1^, Petr Kozlík^3^, Tomáš Křížek^3^, Pavel Ryšánek^2^, Martin Šíma^2^, Ondřej Slanař^2^, Miroslav Šoóš^1^*

^1^ Department of Chemical Engineering, Faculty of Chemical Engineering, University of Chemistry and Technology, Prague, Czech Republic

^2^ Institute of Pharmacology, First Faculty of Medicine, Charles University and General University Hospital in Prague, Prague, Czech Republic

^3^ Department of Analytical Chemistry, Faculty of Science, Charles University, Prague, Czech Republic

^a^ Equally contributing authors

*Corresponding author: Miroslav Šoóš, [miroslav.soos@vscht.cz](mailto:miroslav.soos@vscht.cz)

|  |
| --- |

**Figure-SI 1** XRD diffractogram of prepared CBD nanoparticle samples compared to raw CBD crystals and lecithin.

**Table-SI 1** Pharmacokinetic parameters following administration of reference and tested formulations using a cross-over design. Cmax, AUClast and test/reference ratios are given as geometric means (± 90% CI) and Tmax is given as median (IQR). R1-R11 -reference formulations, T1-T11 -tested formulations. *P<0.05, ** P<0.01. Cmax and AUClast values are dose-normalized to 25 mg/kg.

| **Formulation** | **C_max_ (ng/mL)** | **T/R C_max_ (%)** | **AUC_last_ (ng/mL.h)** | **T/R AUC_last_ (%)** | **T_max_ (min)** |
| --- | --- | --- | --- | --- | --- |
| R1 | 313.8  (186.7-527.2) | N/A | 2335.9  (1569.6-3476.2) | N/A | 298.0  (269.5-331.0) |
| T1 | 812.8  (540.5-1222.0) | 234.2  (131.9-415.6) | 4115.9  (2951.4-5739.8) | 165.6  (112.6-243.4) | 238.0  (159.5-243.5)* |
| R2 | 585.5  (285.0-1202.5) | N/A | 4381.4  (2217.7-8656.2) | N/A | 359.0  (273.0-420.5) |
| T2 | 1186.9  (733.2-1921.2) | 192.6  (92.6-400.5) | 5676.4  (3641.1-8849.3) | 125.4  (91.0-172.8) | 180.0  (179.5-271.5) |
| R3 | 188.5  (130.1-273.2) | N/A | 1553.0  (1061.6-2271.9) | N/A | 357.0  (207.5-421.5) |
| T3 | 888.7  (598.7-1319.3)** | 455.9  (279.6-743.5) | 4357.8  (3486.5-5446.9)** | 291.1  (182.2-465.2) | 176.0  (117.0-240.5) |
| R4 | 466.6  (271.1-802.9) | N/A | 2487.7  (1643.1-3766.4) | N/A | 180.0  (125.0-241.5) |
| T4 | 844.5  (563.6-1265.4) | 193.5  (99.0-378.3) | 3370.6  (2746.9-4135.8) | 142.3  (93.6-216.4) | 122.0  (121.0-152.0) |
| R5 | 587.5  (390.5-883.9) | N/A | 3286.9  (2425.2-4454.7) | N/A | 303.0  (256.5-303.0) |
| T5 | 777.0  (595.2-1014.3) | 132.2  (72.7-241.3) | 3371.8  (2916.6-3898.0) | 102.6  (63.2-166.5) | 213.5  (138.5-332.8) |
| R6 | 172.9  (72.3-413.9) | N/A | 1393.3  (619.0-3136.4) | N/A | 296.0  (239.5-333.5) |
| T6 | 359.1  (222.7-579.3) | 187.1  (49.5-707.8) | 2514.1  (1885.4-3352.3) | 171.5  (54.2-543.0) | 297.0  (239.0-330.0) |
| R7 | 155.3  (118.2-204.1) | N/A | 1058.1  (754.31-1484.2) | N/A | 361.5  (315.8-450.8) |
| T7 | 457.1  (290.0-720.5)* | 294.3  (156.4-553.7) | 2146.3  (1478.3-3116.1) | 202.8  (98.1-419.6) | 186.0  (180.0-273.0) |
| R8 | 301.1  (191.9-472.5) | N/A | 1306.6  (907.6-1881.0) | N/A | 129.0  (120.5-210.0) |
| T8 | 468.6  (363.0-604.9) | 149.8  (75.3-298.1) | 2275.2  (1857.6-2786.6) | 165.5  (98.9-277.2) | 182.0  (155.5-242.0) |
| R9 | 217.4  (118.4-399.2) | N/A | 1612.2  (1016.5-2556.9) | N/A | 247.0  (211.5-308.0) |
| T9 | 437.2  (334.3-571.8)* | 208.8  (112.3-388.4) | 2232.7  (1736.2-2871.2) | 139.5  (82.7-235.4) | 128.0  (123.5-180.5)* |
| R10 | 738.7  (473.6-1152.1) | N/A | 4706.0  (2969.8-7457.2) | N/A | 363.0  (304.0-366.5) |
| T10 | 599.0  (405.2-885.3) | 76.0  (47.8-120.9) | 3136.3  (2461.7-3995.7) | 66.0  (36.7-118.7) | 300.0  (181.0-391.5) |
